# Quantitative muscle MRI captures early muscle degeneration in calpainopathy

**DOI:** 10.1038/s41598-022-23972-6

**Published:** 2022-11-16

**Authors:** Johannes Forsting, Marlena Rohm, Martijn Froeling, Anne-Katrin Güttsches, Nicolina Südkamp, Andreas Roos, Matthias Vorgerd, Lara Schlaffke, Robert Rehmann

**Affiliations:** 1 Department of Neurology, BG-University Hospital Bergmannsheil, Ruhr-University Bochum, Bürkle-de-la-Camp-Platz 1, 44789 Bochum, Germany; 2 Heimer Institute for Muscle Research, BG-University Hospital Bergmannsheil, Bochum, Germany; 3 Department of Radiology, University Medical Centre Utrecht, Utrecht, The Netherlands; 4 Department of Neuropediatrics, University Hospital Essen, Duisburg-Essen University, Essen, Germany

**Keywords:** Neuromuscular disease, Diagnostic markers

## Abstract

To evaluate differences in qMRI parameters of muscle diffusion tensor imaging (mDTI), fat-fraction (FF) and water T2 time in leg muscles of calpainopathy patients (LGMD R1/D4) compared to healthy controls, to correlate those findings to clinical parameters and to evaluate if qMRI parameters show muscle degeneration in not-yet fatty infiltrated muscles. We evaluated eight thigh and seven calf muscles of 19 calpainopathy patients and 19 healthy matched controls. MRI scans were performed on a 3T MRI including a mDTI, T2 mapping and mDixonquant sequence. Clinical assessment was done with manual muscle testing, patient questionnaires (ACTIVLIM, NSS) as well as gait analysis. Average FF was significantly different in all muscles compared to controls (*p* < 0.001). In muscles with less than 8% FF a significant increase of FA (*p* < 0.005) and decrease of RD (*p* < 0.004) was found in high-risk muscles of calpainopathy patients. Water T2 times were increased within the low- and intermediate-risk muscles (*p* ≤ 0.045) but not in high-risk muscles (*p* = 0.062). Clinical assessments correlated significantly with qMRI values: QMFM vs. FF: r = − 0.881, *p* < 0.001; QMFM versus FA: r = − 0.747, *p* < 0.001; QMFM versus MD: r = 0.942, *p* < 0.001. A good correlation of FF and diffusion metrics to clinical assessments was found. Diffusion metrics and T2 values are promising candidates to serve as sensitive early and non-invasive methods to capture early muscle degeneration in non-fat-infiltrated muscles in calpainopathies.

## Introduction

Limb Girdle Muscular Dystrophy (LGMD) R1 is a recessive inherited disease caused by mutations in the *Calpain3* (*CAPN3)* gene which encodes for Ca^2+^-activated intracellular cysteine protease^1^. CAPN3 has been shown to have proteolytic and non-proteolytic functions in skeletal muscles. It may play a role in the maintenance and integrity of sarcomere structure and regulates sarcomeric protein turnover^2^. Clinical presentation of LGMDR1 is characterized by a progressive weakness of limb girdle muscles with variability in disease onset and severity related to the mutation type and gender^3^: Null mutations lead to a more severe phenotype than missense mutations^4^. Recently, autosomal-dominant LGMD patients with calpainopathy were reported (LGMDD4)^5,6^. Patients face difficulties walking, and frequently have a complete loss of ambulance over the years^7^. LGMDR1 is the most common form of LGMD in Europe with a prevalence of 1:15.000–1:150.000^8^. In recent years, different therapeutic options for LGMD have been investigated pre-clinically, varying from immunomodulation to genetic treatments^4^. To evaluate these new therapeutic options, the development of non-invasive surrogate biomarkers is an important complement to patient-specific outcomes, clinical evaluation, and electrophysiology. Quantitative MRI (qMRI) is a promising non-invasive tool in the evaluation of neuromuscular diseases (NMD)^9,10^. Advanced qMRI techniques like Dixon fat-fraction (FF) imaging, quantitative T2 measures or diffusion tensor imaging (DTI) can provide quantitative variables which have shown to be more precise and reliable than semiquantitative rating by visual inspection^11^. Furthermore, they offer additional information about the underlying pathophysiology^12,13^. Dixon sequences enable quantifying and monitoring of fat infiltration in muscles which correlate strongly with clinical assessments^14^. An increase of FF has been shown to precede clinical deterioration of muscle function in different longitudinal studies^15^. Quantitative T2 measures have been associated with myoedema, inflammation and fat infiltration while changes in DTI derived values like fractional anisotropy (FA) and mean diffusivity (MD) reflect denervation, inflammation, metabolic disorders, and injuries^14,15^. Changes in T2 and diffusion metrics can precede fat infiltration shown by Dixon FF in neuromuscular diseases (NMD)^16,17^. While those techniques have been applied successfully to a wide range of NMD like Late Onset Pompe Disease (LOPD) or Spinal Muscular Atrophy (SMA), qMRI information in calpainopathy is sparse^17,19^. MRI studies using classic T1- and T2-weighted images have identified a typical pattern of leg muscle involvement with accentuated fat infiltration of adductors and semimembranosus muscle in thigh muscles while in calf muscles medial gastrocnemius and soleus muscle are mainly affected^19,20^. Arrigoni et al. showed correlation of clinical scores and qMRI values (Dixon fat-fraction) in thigh muscles of eleven patients with genetically confirmed LGMDR1 which have been confirmed by a recent study with a heterogeneous cohort including five patients with LGMDR1^21,22^.

However, information regarding a comprehensive set of both thigh and calf muscles has not yet been published. Therefore, this study aimed to assess a pattern by quantitative imaging parameters of leg muscles in patients with genetically confirmed calpainopathy and to correlate FF, T2 time and DTI parameters to a clinical assessment. Additionally, we aimed to analyse the role of diffusion metrics and T2 to capture early muscle degeneration in (yet) non-fat-infiltrated, normal-appearing muscles of calpainopathy patients. We hypothesized that those qMRI values would be altered at first in muscles with a high risk of fatty infiltration over the disease course, based on previous studies regarding muscle involvement in calpainopathies ^18,20,23^, followed by muscles with intermediate and low risk. We, therefore, classified the low-fat thigh and calf muscles in three risk groups.

## Methods

### Study population

In total, 19 individuals with genetically and/or histologically confirmed calpainopathy (LGMD R1/D4) (10 females, aged 25–69 years; mean age 39.8 years, 9 males; aged 26–56 years; mean age 36.2 years) and 19 age- and gender-matched healthy volunteers (10 females, aged 26–60 years; mean age 39.2 ± 12.6 years) participated in this study. Clinical information of calpainopathy patients is given in Table 1. In all individuals with only a mono-allelic mutation in the genetic testing, diagnosis of calpainopathy was proven by myopathic changes in muscle biopsy specimen and reduced calpain-protein abundance (investigated by immunoblotting). Exclusion criteria for healthy volunteers were medical history of neuromuscular diseases (NMD) and injuries in lower extremity within 12 months prior to study enrolment.

**Table 1:** Demographic and clinical data of patients with calpainopathy.

Patient	Sex	Nucleotide changes in *CAPN3*	Age (years)	Age at onset (years)	Disease duration (years)	QMFM	6-MWT (m)
1	Male	c.550delA (homozygous)*	33	11	22	6	–
2	Male	c.598_612del; c.2393C > A*	37	10	27	10	–
3	Male	c.706G > A; c.2242C > T*	31	3	28	21	285
4	Male	c.550delA; c.1657G > A	25	15	10	21	270
5	Male	c.493T > C; c.872T > C*	56	42	14	38	410
6	Male	c.550delA*	29	14	15	13	Few steps
7	Male	c.966T > A; c.1069C > T	55	13	42	11	–
8	Male	c.717delT (homozygous)*	26	3	23	7	–
9	Male	c.245C > T; c.1355-1G > C	34	6	28	10	–
10	Female	c.550delA; c.2036-2037delCA	25	13	12	44	396
11	Female	c.1746-20C > G	44	23	21	51	612
12	Female	c.966T > A; c.1069C > T	57	25	22	34	275
13	Female	c.550delA; c.2393C > A*	50	33	17	33	278
14	Female	c.2243G > A (homozygous)	33	14	19	22	200
15	Female	c.550delA, del Exon 13–15	27	11	16	17	Few steps
16	Female	c.598_612del*	55	43	12	40	453
17	Female	c.854delA, c.1607C > T	31	12	19	11	Few steps
18	Female	c.550delA; c.2440C > A*	34	16	18	11	Few steps
19	Female	c.598_612del; c1292T > C	68	48	20	29	252

*Diagnosis confirmed via muscle biopsy and reduced Calpain-3 protein abundance investigated by immunoblotting.

The institutional and committee for approving the experiments was the ethics committee of the Faculty for Medicine of the Ruhr University Bochum (Ruhr University Bochum No. 15-5281). The study was performed in accordance with the guidelines of the ethics committee and line with the Declaration of Helsinki (DVH), the relevant national and international recommendations according to Good Clinical Practice (GCP), §7 Health Professions Act North Rhine-Westphalia and §15 of the Professional Code of the Medical Association of Westphalia-Lip. Informed consent was obtained from all participants.

### Clinical assessments

Muscle strength was evaluated using both, the Medical Research Council (MRC) scale and Quick Motor Function Test (QMFT) by an experienced clinician (JF: 5 years of experience, RR: 8 years of experience, AKG: 16 years of experience)^24^. Hip flexion, knee flexion, knee extension, ankle dorsiflexion, and ankle plantarflexion were assessed with a Chatillon Dynamometer® (Chatillon DFE II Dynamometer, Chatillon Force Measurement, AMETEK, USA) for force measurements. Daily life activities were enquired using the ACTIVLIM and the NSS^24,25^. An experienced medical technical assistant performed the following tests in ambulant individuals: the 6-MWT, time to walk 10 m, and timed up- and-go test.

### MRI acquisition and sequences

The participants laid in a feet-first supine position. Cushions were used to support participants’ knees and sandbags placed around their feet to prevent motion. Scans were obtained using a Philips 3.0T Achieva MR system and a 16CH Torso XL coil. The thigh region from hip to knee was split into two axial fields of view (FOV) of 480 × 276 × 150 mm^3^ along the z-axis with a 30 mm overlap and the proximal edge positioned in the crotch. The calf region was scanned with one axial FOV of 480 × 276 × 150 mm^3^. The proximal edge of the FOV was positioned 60 mm below the tibial plateau perpendicular to the tibial bone. The protocol consisted of a 4-point Dixon sequence (voxel size 1.5 × 1.5 × 6.0 mm^3^; TR/TE 210/2.6, 3.36, 4.12, 4.88 ms; flip angle 8°, SENSE: 2), a multi‐echo spin‐echo (MESE) sequence for quantitative water mapping including 17 echoes and Cartesian k‐space sampling (voxel size 3.0 × 3.0 × 6.0 mm^3^; TR/TE 4598/17 × ∆7.6 ms; flip angle 90/180°, SENSE: 2), and a diffusion-weighted spin-echo EPI (voxel size 3.0 × 3.0 × 6.0 mm^3^; TR/TE 5000/57 ms; SPAIR/SPIR fat suppression; SENSE: 1.9; 42 gradient directions with eight different b-values (0–600 s/mm^2^)^26^. A noise scan was obtained using the same imaging parameters as the DWI, but without RF power and gradients (only acquisition channels open). Scanning time per stack (each FOV) was approximately 12 min.

### Data pre-processing

Data were pre-processed as described before using QMRITools (www.qmritools.com)^26^. In brief, the diffusion data were denoised using a PCA method^27^. To correct for subject motion and eddy currents both legs were registered separately. Then the tensors were calculated by taking IVIM into account and using an iWLLS algorithm^27,28^. The IDEAL method was used for the Dixon data considering a single T2* decay and resulting in a separated water and fat map^30^. The derived water maps were used for the manual segmentation. The T2‐mapping data were analysed using an extended phase graph (EPG) fitting approach^31^.

### Muscle segmentation and tractography

Eight thigh muscles (vastus lateralis, vastus medialis, rectus femoris, semimembranosus, semitendinosus, biceps femoris, sartorius, and gracilis) and seven calf muscles (gastrocnemius medialis and lateralis, soleus, tibialis anterior, peroneus, extensor digitorum and tibialis posterior) were first segmented in patients and controls using an automated segmentation tool and subsequently optimized by an experienced rater (JF) in both legs^32^. The rater checked the automated segmentation results and manually corrected the muscle shape if necessary. Automated segmentation was not precise enough in patients due to loss of muscle structure in fatty infiltrated muscles. Patient muscles were segmented manually on all 25 acquired slices of the Dixon water images (3D-slicer 4.4.0, https://www.slicer.org)^33^.

The segmentations were then registered to T2 and DTI data to correct for small motions between sequences and image distortions using sequential rigid and b-spline transformations (elastix, https://elastix.lumc.nl^34^. Average values over all slices of water-T2 time and proton density fat fraction (FF) were obtained. SNR was calculated as the local average signal divided by the local noise sigma^35^. For analysis of diffusion data, the pre-processed diffusion data were divided based on the muscle segmentation. Secondly, whole muscle tractography was performed within each diffusion muscle volume. (MRIToolkit)^36^. The following fiber tracking stop parameters were used: maximum angle 15°, step size 1.5 mm, FA range 0.1–0.6^35,36^. The DTI parameters fractional anisotropy (FA), mean diffusivity (MD), axial diffusivity (λ_1_) and radial diffusivity (RD) were extracted for each individual muscle using tract-based sampling.

### Statistical analysis

FF was compared between LGMD patients and healthy controls in a general linear model with patient/control, body side, and muscle (to control degrees of freedom for multiple test points per subject) as fixed factors for all leg muscles.

For the analysis of the diffusion metrics and T2 times, we chose a disease-specific approach in terms of muscle involvement, based on the degree of muscle fat infiltration shown by individual muscle FF in this study and literature review^18^. We chose to analyse qMRI changes in different groups of muscles that have a different risk of degeneration and not in a patient/control approach.

We identified the three following muscle groups with different risks of fat infiltration in calpainopathies^20^:

I. high risk: biceps femoris, semimembranosus, semitendinosus, gastrocnemius medialis and soleus
II. intermediate risk: vastus medialis, vastus lateralis, rectus femoris, sartorius, gracilis, gastrocnemius lateralis and peroneal group
III. low risk: extensor digitorum, tibialis anterior and tibialis posterior

To assess differences in diffusion metrics and T2 time between low-fat muscles of controls and LGMD patients, the cut-off value for low-fat muscles was defined as: highest mean fat fraction in healthy controls + 1 SD (~ 8%)^17^.

Additionally, muscles with low diffusion data quality, which was defined by a SNR of lower than 10 before denoising, were excluded for analysis of diffusion parameters^39^. After performing Levene’s test for equality of variances, two-sided t-tests for independent samples between LGMD and control group were conducted to evaluate the following hypotheses for muscles with < 8% FF:

I. In high-risk muscles we hypothesize that the known fiber atrophy and myocellular damage in calpainopathies^40^ could lead to an increase of FA, a decrease of MD and RD and an increase in T2.
II. In intermediate-risk muscles, there may be significant differences in T2 and diffusion metrics between the patient and control group.
III. In low-risk muscles no significant changes of T2 and diffusion metrics between study groups can be detected.

To evaluate correlations between clinical assessments and qMRI values mean FF, FA, MD, and T2 of all thigh and calf muscles were correlated to the 6-MWT, time to walk 10 m, and timed up-and-go test using Pearson’s correlation coefficients. Furthermore, mean qMRI values of thighs and calves were correlated to the results of ACTIVLIM, NSS, and QMFM using Spearman’s rank correlation coefficients. Additionally, the qMRI values of the corresponding muscle groups were correlated to the force measurements by dynamometry and MRC scale, i.e., quadriceps muscle and knee extension.

All statistical analyses were performed using IBM SPSS V28. The significance level for all tests was set at *p* < 0.05.

## Results

**Figure 1:**
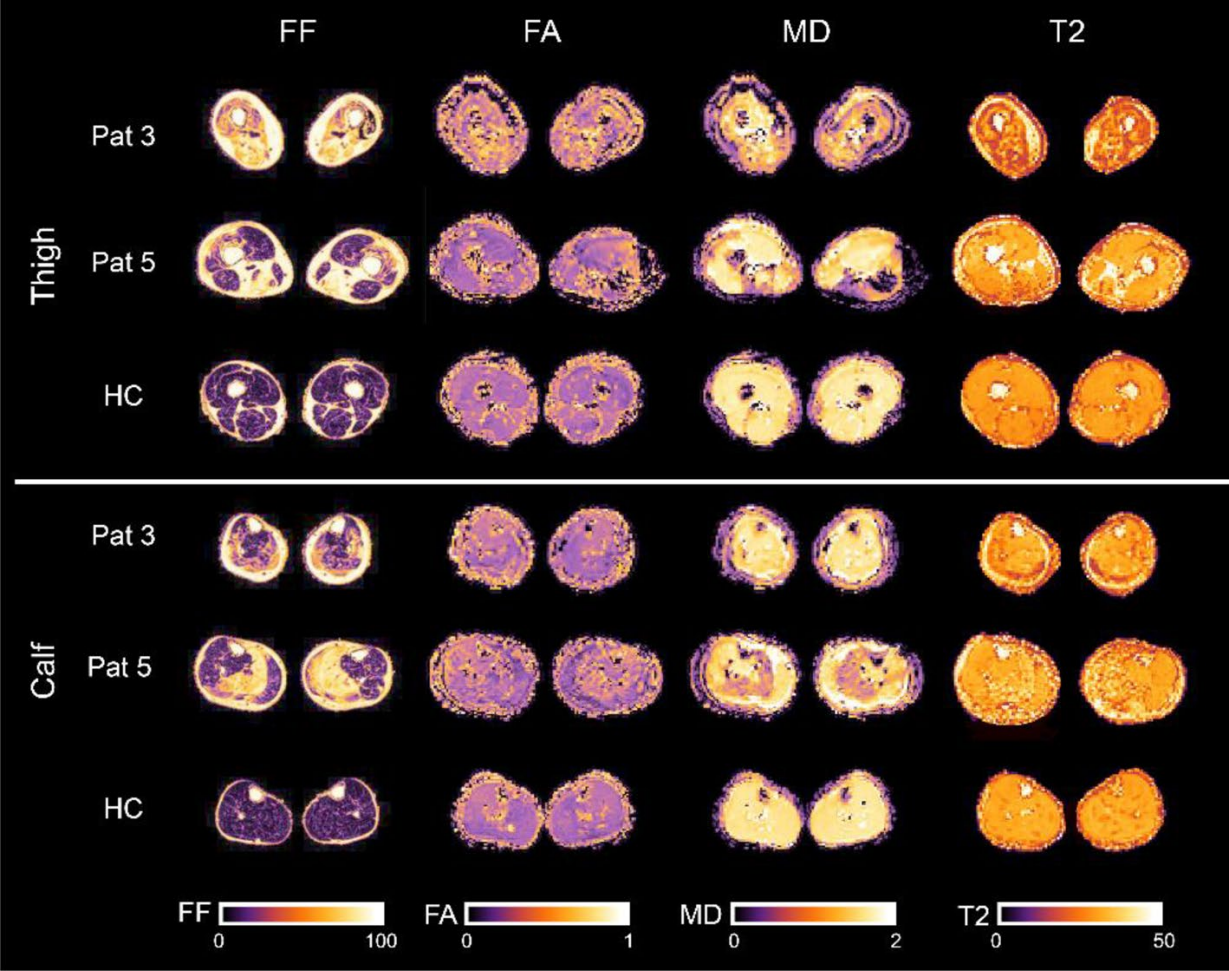
Example images of the applied MRI sequences. mDixon fat fraction (FF), fractional anisotropy (FA), mean diffusivity (MD) and T2 maps for thigh and calf muscles of two representative LGMD patients, and a healthy control (HC).

All scans and clinical assessments were successfully performed. Example images of the applied MRI sequences are shown in Fig. 1.

**Figure 2:**
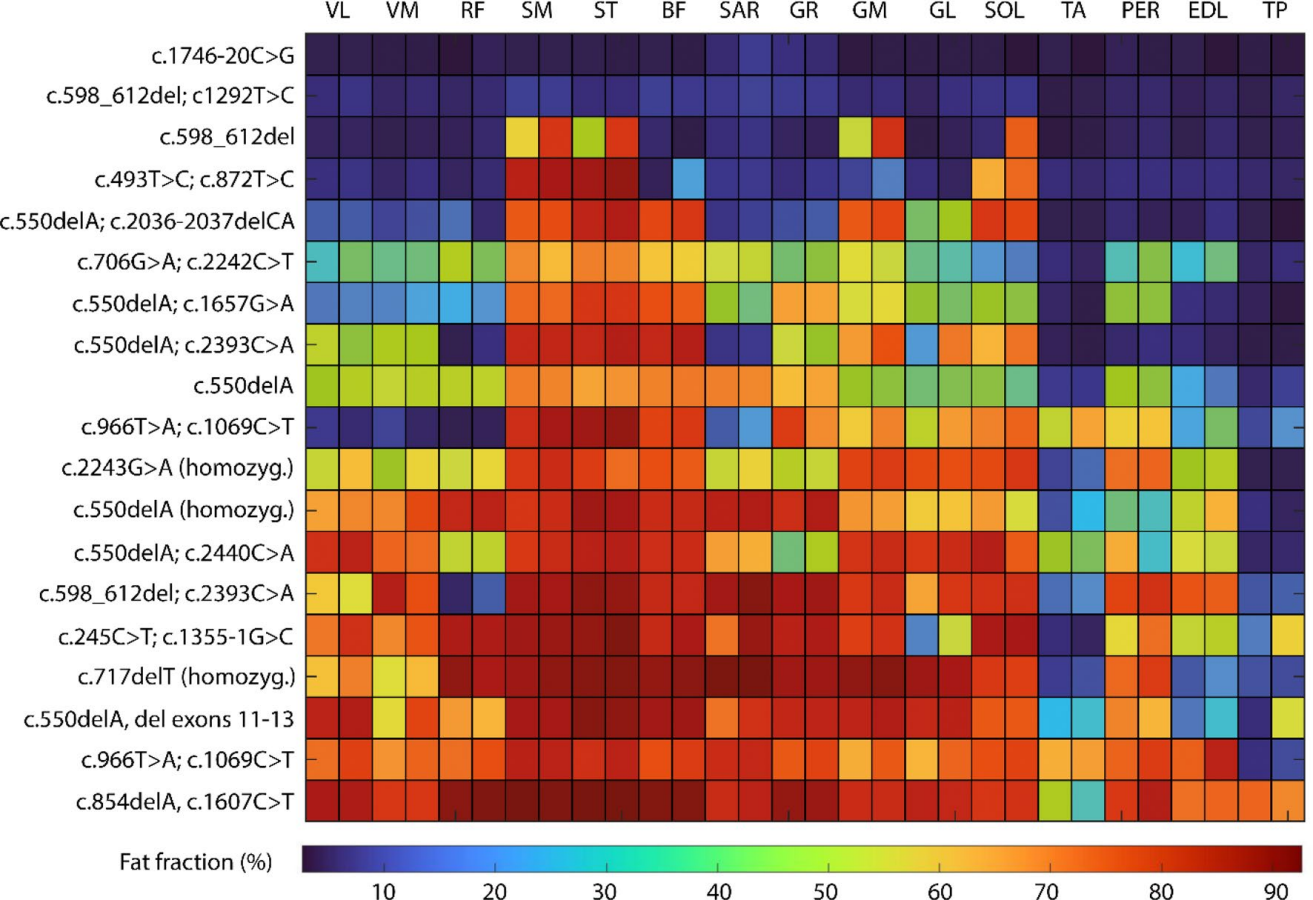
Overview of fat infiltration and mutations in calpainopathy patients. *VL* vastus lateralis, *VM* vastus medialis, *RF* rectus femoris, *SM* semimembranosus, *ST* semitendinosus, *BF* biceps femoris, *SAR* Sartorius, *GR* gracilis, *GM* gastrocnemius medialis, *GL* gastrocnemius lateralis, *SOL* soleus, *TA* tibialis anterior, *PER* peroneal group, *EDL* extensor digitorum longus, *TP* tibialis posterior.

**Figure 3:**
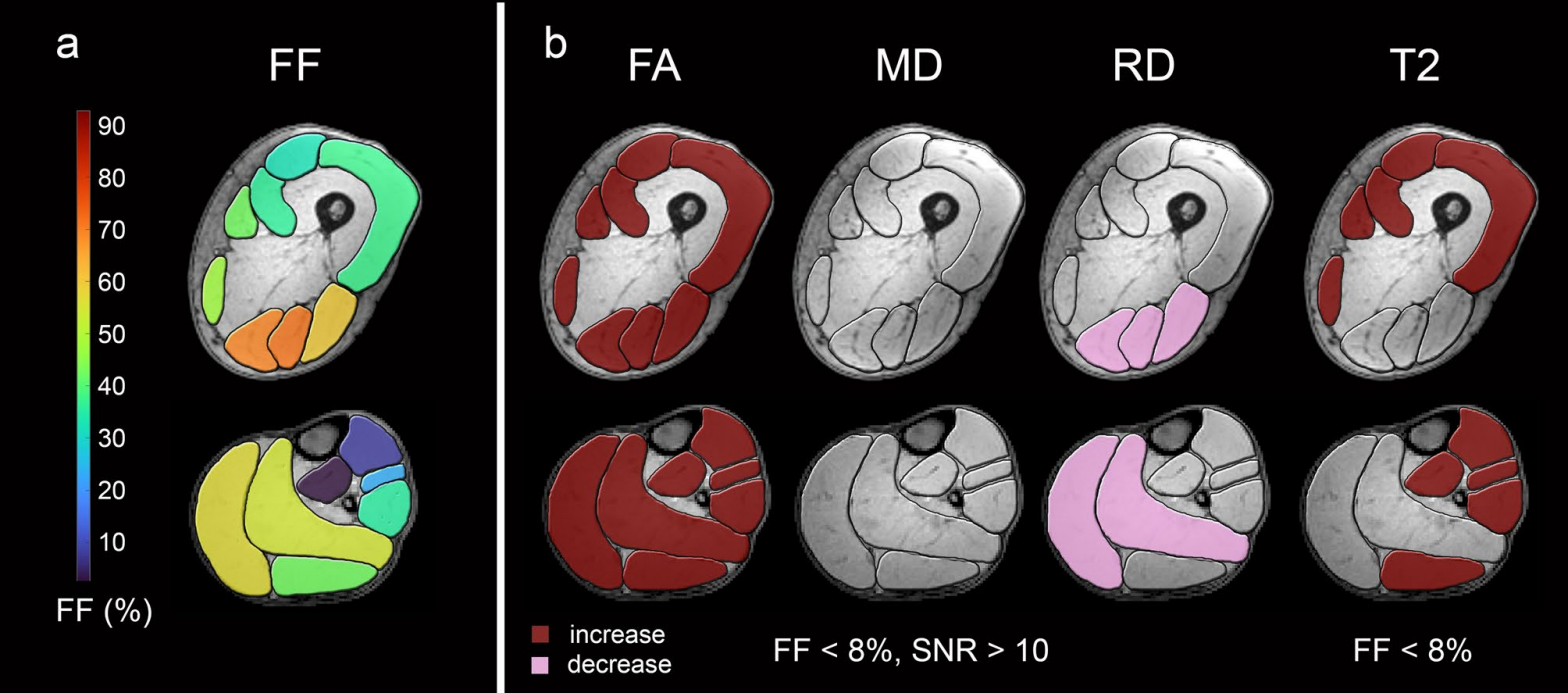
qMRI data in low-fat muscles. Overview of mean fat fractions of all LGMD patients in thigh and calf muscles (**a**). High-risk muscles are coloured in yellow and orange, intermediate-risk muscles are coloured in green and low-risk muscles are coloured in blue. Muscle groups with significant differences of FA and MD in muscles with FF < 8% and SNR > 10 and T2 in muscle groups with FF < 8% between study groups are coloured in red (**b**) (increase/decrease in patient group: burgundy / pink).

The average FF was significantly different in all patient muscles than in controls (Main effect: *p* < 0.001; Bonferroni-corrected post-hoc t-tests for each muscle: *p* ≤ 0.047). The mean FF per muscle of LGMD patients is displayed in Fig. 2. The tibialis posterior was less affected in LGMD patients, while semimembranosus and semitendinosus showed the highest FF. Average mean FF values per muscle of all LGMD patients are illustrated in Fig. 3.

When comparing diffusion metrics between patient and control group in muscles with less than 8% FF and SNR higher than 10, a significant increase of FA and decrease of RD was found in high-risk muscles of LGMD patients (see Table 2 and Fig. 3). MD showed a nonsignificant decrease in LGMD group (*p* = 0.121). In low- and intermediate-risk muscles, a significant increase of FA was observed, while the other diffusion metrics showed no differences in comparison to the control group. When comparing T2 between study groups we found an increase in all previously defined muscle groups which was significant in the low- and intermediate-risk groups (*p* ≤ 0.045) but not for high-risk group (*p* = 0.062).

**Table 2:** Mean differences and p-values for two-sided t-tests for independent samples between patients and controls for the different muscle groups.

		High risk muscles	Intermediate risk muscles	Low risk muscles
**FF < 8%**				
*n (LGMD/CON)*		24/189	71/263	60/114
T2 ms	∆ mean	0.388	0.409	0.311
	*p value*	0.062	< 0.001*	0.045*
**FF < 8% & SNR > 10**				
*n (LGMD/CON)*		23/188	69/252	57/114
FA	∆ mean	0.018	0.016	0.012
	*p value*	0.005*	< 0.001*	0.003*
MD (10^−3^ mm^2^/s)	∆ mean	− 0.032	− 0.014	0.016
	*p value*	0.121	0.458	0.346
Λ_1_ (10^−3^ mm^2^/s)	∆ mean	− 0.010	0.013	0.047
	*p value*	0.759	0.562	0.057
RD (10^−3^ mm^2^/s)	∆ mean	− 0.042	− 0.027	0.001
	*p value*	0.004*	0.132	0.957

Significant results are marked with an *. *p* < 0.05 was defined as statistically significant.

**Figure 4:**
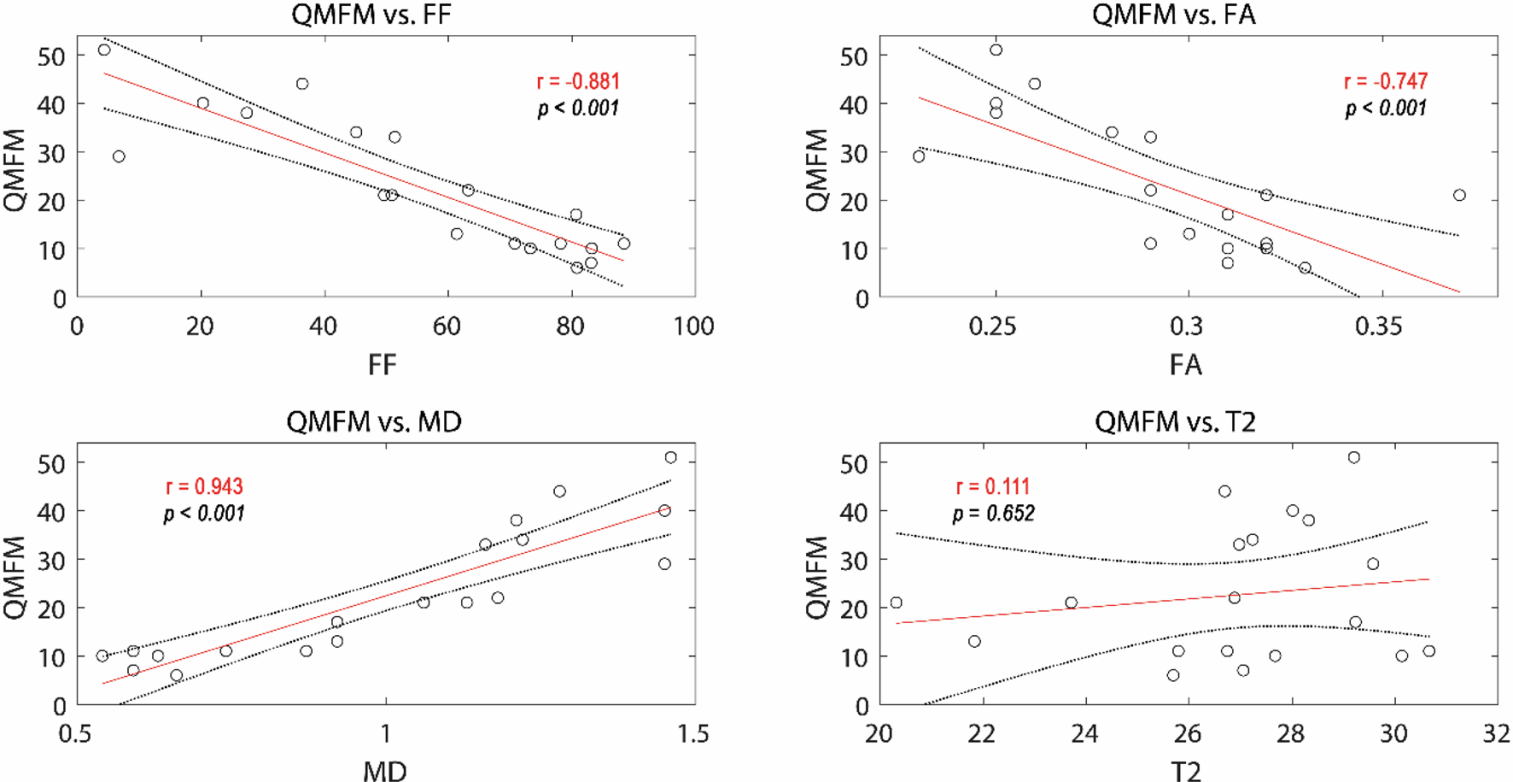
qMRI data and QMFM correlations. Spearman rank correlation coefficients (r) of qMRI values fat fraction (FF), fractional anisotropy (FA), mean diffusivity (MD), and T2 time of all thigh muscles to Quick Motor Function Measure (QMFM). Regression lines are coloured in red, 95%-confidence intervals are coloured in black.

Correlations between clinical assessments and qMRI values in LGMD patients are displayed in Tables 3, 4 and Supplement Table S1. A significant correlation between 6-MWT and FF of thigh muscles was observed (r = − 0.691, *p* < 0.05). For the other qMRI values weak to moderate correlations were found (see Table 3). Timed-up-and-go showed a strong correlation to FA and T2 in thigh muscles (*p* < 0.01), while 10 m walking-time correlated strongly with FF, FA and MD in thigh muscles and FF and FA in calf muscles. QMFM showed strong correlation with FF, FA, and MD in the thigh and a moderate to strong correlation with those parameters in calf muscles (*p* < 0.01; see Fig. 4). ACTIVLIM showed higher correlations to FF, FA and MD than NSS (|r|≥ 0.631 vs. |r|≥ 0.372; see Table 4). No significant correlations of questionnaires and QMFM to T2 were found. The force measurements of knee extensors, knee flexors and dorsi extensors showed moderate to good correlation to FF, FA and MD.

**Table 3:** Overview of Pearson correlation coefficients (r) for qMRI parameters fat fraction (FF), fractional anisotropy (FA), mean diffusivity (MD), and T2 relaxation time (T2) and clinical outcome measures in ambulant calpainopathy patients.

	Thigh muscles				Calf muscles			
	FF %	FA	MD (10^−3^ mm^2^/s)	T2 ms	FF %	FA	MD (10^−3^ mm^2^/s)	T2 ms
6-MWT (m)	− 0.691*	− 0.601	0.518	0.380	− 0.614	− 0.357	0.369	0.501
Timed-up-and-go (s)	0.651*	0.707*	− 0.465	− 0.771**	0.461	0.768**	− 0.322	− 0.808**
10 m walking time (s)	0.792**	0.861**	− 0.679**	− 0.201	0.745**	0.816**	− 0.632*	− 0.301

*p < 0.05, **p < 0.01. *m* meters, *s* seconds.

**Table 4:** Overview of Spearman rank correlation coefficients for qMRI parameters fat fraction (FF), fractional anisotropy (FA), mean diffusivity (MD), and T2 relaxation time (T2) and clinical outcome measures in calpainopathy patients.

	Thigh muscles				Calf muscles			
	FF %	FA	MD (10^−3^ mm^2^/s)	T2 ms	FF %	FA	MD (10^−3^ mm^2^/s)	T2 ms
ACTIVLIM	0.779**	0.477*	− 0.732**	− 0.090	0.666**	0.556*	− 0.788**	− 0.201
NSS	− 0.582**	− 0.356	0.589**	− 0.150	− 0.565*	− 0.386	0.694**	0.047
QMFM	− 0.884**	− 0.645**	0.831**	0.102	− 0.639**	− 0.679**	0.842**	0.268

*p < 0.05, **p < 0.01.

The highest correlations were found for FF (see Supplement Table S1).

## Discussion

In recent years, qMRI has shown to be a useful tool in diagnostic management and disease monitoring of NMD. The advantages of the combination of MRI measurements and clinical assessments in the disease monitoring of patients with LGMDR1 were first shown by Fardeau and colleagues^41^. In recent years, several semiquantitative MRI studies identified a pattern of fat distribution in calpainopathies showing a predominant involvement of the hamstrings and the soleus and gastrocnemius medialis^19,20,40^. Using Dixon FF, we confirmed those observations in a cohort of 19 calpainopathy patients presented in this study. As Barp et al. described before, relative sparing of quadriceps, sartorius, and gracilis in the thigh and anterolateral compartment of the calf was identified^20^. The least fat-infiltrated muscle was the tibialis posterior displaying a fat fraction of lower than 20% even in highly affected individuals (see Fig. 2)^42^. FF is known to correlate with MFM and force measurements by MRC scale in LGMDR1 and has shown superior sensitivity over standard functional evaluation^21,41^. In this study, significant moderate to strong correlations to different clinical outcome measures were observed not only for FF but also for diffusion metrics FA and MD. Since ACTIVLIM correlated more strongly to FF than NSS, ACTIVLIM questionnaire might be more suitable in monitoring of LGMD. This may be explained by the fact that several items in NSS address orofacial weakness which in LGMD is usually not observed. The highest correlation was identified between FF in thigh muscles and the QMFM suggesting that although being a general assessment QMFM can best reflect degree of fat infiltration. In contrast to the findings of Arrigoni and colleagues, in a cohort of 11 patients with LGMDR1, in our study moderate to strong correlations of diffusion metrics FA and MD to clinical outcome measures were observed^22^.

These different results may be explained by methodological reasons: We used a different post-processing algorithm to obtain our DTI data for the whole muscle from tractography. This approach might be more sensitive to detect degenerative changes, as diffusion data can be analysed from the whole muscle volume. In contrast, Arrigoni et al. used an ROI-based method in which the different muscle compartments were segmented on a single slice only. Even with careful placement of the ROI, this method may over- or underestimate DTI values in the case of inhomogeneous muscle degeneration. This can be particularly important when correlating to clinical as well as functional data, as the whole muscle is important for these parameters. Furthermore, in our study all muscles were segmented separately to avoid inclusion of muscular fascia and connective tissue. Correlations of FA and MD to clinical assessments have been described before and although those findings must be interpreted with caution due to expected changes of diffusion metrics in highly fat-infiltrated muscles, it has been shown that diffusion metrics can provide additional information on muscle status^18,37^. Arrigoni et al. suggested that diffusion metrics and T2 values should only be examined in preserved muscles^22^.

Williams et al. analysed the effects of fat infiltration in muscle on DTI parameters and concluded that at least 76% of healthy muscle tissue should be present in a muscle to correctly calculate diffusion parameters, otherwise FA is overestimated^44^.

However, Otto et al. were able to show in a model for fat infiltration in muscle tissue that with adequate fat suppression only the water signal of the muscle is used for DTI and sufficiently sensitive results for muscle-specific DTI values can also be achieved in fat-infiltrated muscle^19^. We used the same method of fat suppression for our study. To avoid possible confounding effects of fat infiltration in this study diffusion metrics and T2 values were only evaluated in low-fat muscles. Fatty infiltration in myopathies is not just a confounder, it is also the visible aspect of advanced and non-reversible muscle damage. Therefore, sensitive qMRI biomarkers should capture microstructural changes before fatty infiltration occurs as those would ideally capture a stage where therapies could still be effective^45^. As described, calpainopathies show a typical disease pattern with predominant involvement of hamstrings, gastrocnemius medialis and soleus. Consequently, differences in diffusion metrics and T2 times in this study were assessed in muscle groups that were previously defined as dependent on the risk of fat-infiltration. A significant increase of FA was found in all muscle groups of patients while MD remained unchanged. This may reflect fiber atrophy which is supported by a significant decrease of RD found in this study. A reduction in RD is a sign of a reduced fiber diameter^46^. An increase of FA with an accompanying decrease of MD was also interpreted as a sign of fiber atrophy in NMD^19^. However, Berry et al. showed in simulations that FA changes are more pronounced and precede MD changes with a decreasing muscle fiber size which may explain that no significant differences of MD were found in this study^47^.

A potentially more sensitive approach to measuring muscle fibre diameter was recently published by Tan et al. Using tissue-specific multi-compartment modelling obtained from an orientation invariant dictionary, which models muscle fibres as cylinders with radial constrained diffusivity, myofiber diameter can be measured in µm (AFD—Apparent Fiber Diameter)^48^.

In patients with healthy and denervated shoulder muscles, AFD has been shown to measure myofiber diameter with high sensitivity. The FA increase, RD decrease and unchanged MD shown by Tan et al. in the denervated muscles due to fiber atrophy support that changes of diffusion metrics in non-fat infiltrated muscles of LGMD patients in this study are related to early fiber atrophy. Focal fiber atrophy in calpainopathies has been described in muscle biopsies of still asymptomatic individuals suggesting that fiber atrophy occurs early in course of disease^49^.

Another histopathological finding in muscle biopsy of patients with calpainopathies (but also other muscular dystrophies) is a higher number of eosinophils in comparison to healthy controls which may reflect role of inflammation in pathophysiology^50^. In this study an increase in T2 values in all groups of non-fat infiltrated LGMD muscles was found as an indication of either an inflammatory process or a sign of active muscle degeneration. Correlations of T2 values to clinical assessments were lower than of the other qMRI parameters. Thus, elevated T2 does not translate immediately to clinical function and may precede an impairment of those prior to changes in DTI metrics. However, increase of T2 values can very well distinguish between healthy controls and low-fat muscles of LGMD patients. Therefore, diffusion metrics and T2 values can capture changes in not yet fatty-infiltrated muscles in calpainopathies.

## Limitations

There are some limitations in this study. Due to the early onset of the disease (median disease duration: 19 years) most patients in this study had advanced fat infiltration. Consequently, analysis of low-fat muscles was limited due to a low number of preserved muscles and high level of fat-infiltration of predominantly affected muscles in our cohort. Furthermore, being a cross-sectional cohort study, our study did not reflect the role and changes of qMRI values during disease progression. Longitudinal studies are needed in the future which ideally should include less affected patients.

## Conclusion

Dixon FF values confirm the predominant involvement of hamstrings in the thigh and soleus and gastrocnemius medialis in the calf with relative sparing of anterior muscle group. A good correlation of FF but also diffusion metrics to clinical assessments were found. Diffusion metrics and T2 values are promising candidates to capture early muscle degeneration in non-fat-infiltrated muscles in calpainopathies.

### Supplementary Information

Supplementary Table S1.

## Data Availability

The data that support the findings of this study are not openly available due to sensitivity of human data and to protect patient privacy. The data are available from the corresponding author upon written reasonable request. Any written request will be reviewed by the data protection officer of the University Hospital Bergmannsheil Bochum prior to access.

## Abbreviations

6-MWT: 6-Minute walk test
ACTIVLIM: Questionnaire to measure activity limitations in neuromuscular disorders
CAPN3: Calpain3
FA: Fractional anisotropy
FF: Fat fraction
FOV: Field of view
LGMD: Limb Girdle Muscular Dystrophy
MD: Mean diffusivity
MRC: Medical Research Council
NMD: Neuromuscular diseases
NSS: Neuromuscular symptom score
QMFT: Quick Motor Function Test
qMRI: Quantitative magnetic resonance imaging
RD: Radial diffusivity
SNR: Signal to noise ratio
