# Author Correction: Quantitative muscle MRI captures early muscle degeneration in calpainopathy

**DOI:** 10.1038/s41598-024-61083-6

**Published:** 2024-05-06

**Authors:** Johannes Forsting, Marlena Rohm, Martijn Froeling, Anne-Katrin Güttsches, Nicolina Südkamp, Andreas Roos, Matthias Vorgerd, Lara Schlaffke, Robert Rehmann

**Affiliations:** 1 Department of Neurology, BG-University Hospital Bergmannsheil, Ruhr-University Bochum, Bürkle-de-La-Camp-Platz 1, 44789 Bochum, Germany; 2 Heimer Institute for Muscle Research, BG-University Hospital Bergmannsheil, Bochum, Germany; 3 Department of Radiology, University Medical Centre Utrecht, Utrecht, The Netherlands; 4 Department of Neuropediatrics, University Hospital Essen, Duisburg-Essen University, Essen, Germany

Correction to: *Scientific Reports* 10.1038/s41598-022-23972-6, published online 16 November 2022

The original version of this Article contained an error in Figure 3, where the colour code for ‘increase’ and ‘decrease’, stated within the image, was switched. The original Figure 3 and accompanying legend appear below.

**Figure 3:**
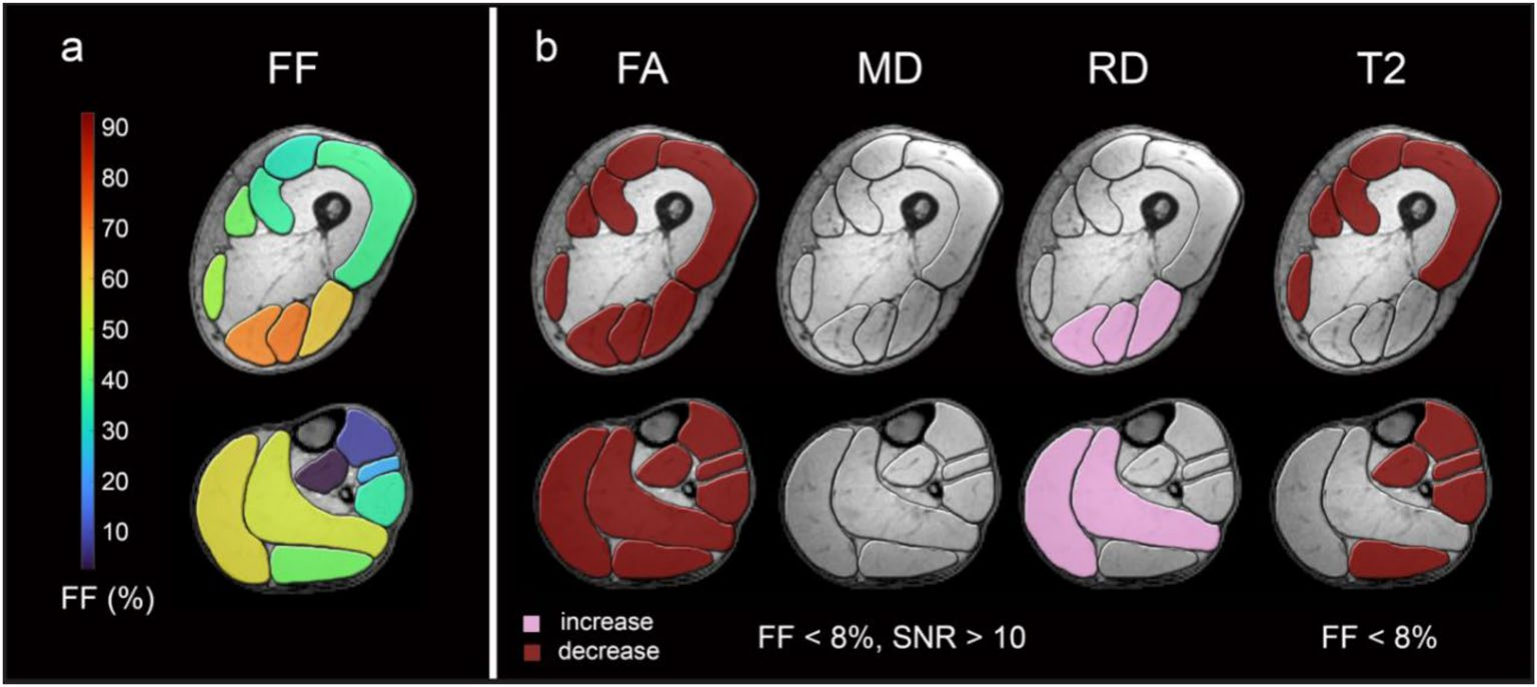
qMRI data in low-fat muscles. Overview of mean fat fractions of all LGMD patients in thigh and calf muscles (**a**). High-risk muscles are coloured in yellow and orange, intermediate-risk muscles are coloured in green and low-risk muscles are coloured in blue. Muscle groups with significant differences of FA and MD in muscles with FF < 8% and SNR > 10 and T2 in muscle groups with FF < 8% between study groups are coloured in red (**b**) (increase/decrease in patient group: burgundy / pink).

The original Article has been corrected.

