# Supplementary material for: Quantitative muscle MRI captures early muscle degeneration in calpainopathy
Source: Sci Rep. 2022 Nov 16;12:19676. doi: 10.1038/s41598-022-23972-6 (PMC9669006; doi:10.1038/s41598-022-23972-6)
Supplement: Supplementary file 1 — Supplementary Table S1. [file 41598_2022_23972_MOESM1_ESM.docx]

|  | **Knee extensors** | | | | **Knee flexors** | | | |
| --- | --- | --- | --- | --- | --- | --- | --- | --- |
|  | MRC | | Dynamometry | | MRC | | Dynamometry | |
|  | Right | Left | Right | Left | Right | Left | Right | Left |
| FF | -0.692** | -0.719** | -0.638** | -0.710** | -0.440 | -0.486* | -0.733** | -0.760** |
| FA | -0.488* | -0.504* | -0.328 | -0.396 | -0.474* | -0.472* | -0.556* | -0.548* |
| MD | 0.505* | 0.567* | 0.402 | 0.526* | 0.459* | 0.502* | 0.415 | 0.454 |
| T2 | 0.560* | 0.626** | 0.431 | 0.494* | 0.054 | 0.004 | 0.406 | 0.383 |
|  | **Dorsal extensors** | | | | **Dorsal flexors** | | | |
|  | MRC | | Dynamometry | | MRC | | Dynamometry | |
|  | Right | Left | Right | Left | Right | Left | Right | Left |
| FF | -0.749** | 0.681** | -0.610** | -0.715** | -0.356 | -0.356 | -0.125 | -0.194 |
| FA | -0.431 | -0.319 | -0.148 | -0.117 | -0.329 | -0.329 | 0.155 | 0.175 |
| MD | 0.682** | 0.558* | 0.539* | 0.549* | 0.296 | 0.296 | -0.038 | -0.086 |
| T2 | 0.225 | 0.167 | 0.257 | 0.212 | 0.030 | 0.030 | 0.291 | 0.296 |

Table S1: Overview of Pearson correlation coefficients between quantitative MRI measures fat fraction (FF), fractional anisotropy (FA), mean diffusivity (MD), and T2 relaxation time (T2) and dynamometry results and Spearman rank correlation coefficients between qMRI values and MRC values in the corresponding muscle groups of calpainopathy patients. *p < 0.05, **p < 0.01
